# The Role of Plant Growth-Promoting Microorganisms (PGPMs) and Their Feasibility in Hydroponics and Vertical Farming

**DOI:** 10.3390/metabo13020247

**Published:** 2023-02-09

**Authors:** Faten Dhawi

**Affiliations:** Agricultural Biotechnology Department, College of Agricultural and Food Sciences, King Faisal University, Al-Ahsa 31982, Saudi Arabia; falmuhanna@kfu.edu.sa

**Keywords:** agriculture, hydroponics, microorganism, vertical agriculture, aquaponics, aeroponics

## Abstract

There are many reasons for the increase in hydroponics/soil-free systems in agriculture, and these systems have now advanced to the form of vertical farming. The sustainable use of space, the reduction in water use compared to soil-based agriculture, the lack of pesticides, the ability to control nutrient inputs, and the implementation of user-friendly technology for environmental control and harvesting are all factors that have made the global market for vertical farming predicted to reach more than USD 10.02 billion by 2027. By comparison, soil-based agriculture consumes 20 times more water, and some agricultural practices promote soil deterioration and cause environmental pollution. Plant growth-promoting microorganisms (PGPMs) have been used extensively in traditional agriculture to enhance plant growth, environmental stress tolerance, and the efficacy of phytoremediation in soil-based farming. Due to the controlled atmosphere in hydroponics and vertical farms, there is strong potential to maximize the use of PGPMs. Here, we review the leveraging of plant growth-promoting microorganism mechanisms in hydroponics and vertical farming. We recommend a synchronized PGPM treatment using a biostimulant extract added to the hydroponic medium while also pre-treating seeds or seedlings with a microbial suspension for aquaponic and aeroponic systems.

## 1. Introduction

The role of plant growth-promoting microorganisms (PGPMs) has been extensively studied in soil-based systems. PGPMs, including plant growth-promoting bacteria (PGPB), arbuscular mycorrhizal fungi (AMF), and rhizobia, increase the production of biomass in plants through synthesizing hormones, fixing nitrogen, and solubilizing phosphate and potassium [1]. Their positive functions include increasing the growth and subsequent metabolic pathways in poor soil with low nutrient levels, and increasing plant tolerance and the phytoremediation ability in polluted soil [2,3,4,5]. Some microorganisms play an additional role as metagenome signatures for some plant species, such as different date palm and millet cultivars [3,6,7,8].

There are multiple physiological mechanisms involved in the growth and development, phytoremediation ability, and tolerance enhancement processes of PGPMs. The interactions between plants and microbes form mutually beneficial relationships where the microbes play a critical role in the plants’ adaptation to a toxic environment and stimulate the growth of the plants, thus augmenting phytoremediation or abiotic stress tolerance [9]. For example, plants support hydrocarbon-degrading microorganisms through their rhizosphere effects; these are helpful for phytoremediation in the root zone [10]. Additionally, the reduction of pollutants in the soil and the ability of PGPMs to increase plant tolerance are based on harboring genes for the mineralization of various inorganic and organic compounds to produce non-toxic products [11].

Currently, the necessity for soil maintenance, the cost of fertilizer and pesticides, and climate challenges have led to an increase in the use of innovative agricultural solutions such as hydroponics and vertical farming [12,13]. Vertical farming is the urban agricultural form of hydroponics [12,13] and offers solutions to reduce land and water use, increase crop yield, and eliminate the use of pesticides and soil fertilizers [14,15]. Thus, vertical farming is considered an improvement in quality and a revolutionary solution for climate change issues [16,17]. However, vertical farming has a limited capacity for crops, as evidenced by the leveraging of this technology to grow specific plants such as microgreens (e.g., arugula, radishes, and bok choy—6%), leafy greens (e.g., lettuce—57%), flowers—10%, tomatoes—16%, and herbs—11% [14,17]. 

Considering that plant growth-promoting microorganisms (PGPMs) have been known for decades to enhance plant health and increase productivity [1,2,3,4,5,6,7,8,9,10,11], the questions are “what is the added value for PGPM incorporation in vertical farming or hydroponics?” and “what is the best mode of use considering a soil-free system?”

**Figure 1:**
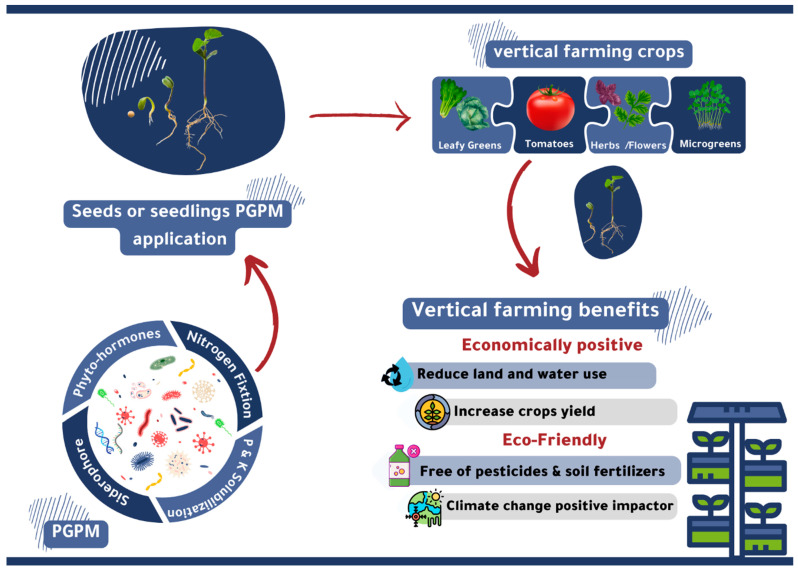
The types of plants used and the economic and environmental feasibility of growth-promoting microorganisms in hydroponics and vertical farming.

Here, we review the literature to date regarding the benefits of the mechanisms of plant growth-promoting microorganisms (PGPMs), as well as highlight the plant–microbial interaction utilization in different types of hydroponics and vertical farming systems (Figure 1).

## 2. The Role of PGPMs in Growth and Productivity

Microorganisms are ubiquitous in nature and they can interact with any other organisms, hence they can have both negative and positive influences, although they are most beneficial and symbiotic with plants [18]. Plants associated with microbial diversity are a product of ecological and evolutionary events [18,19]. Plants and microbes are intimately related to one another and cannot be considered separate components of the ecosystem, hence they are described as eco-holobionts. Microorganisms form a complex community with plants to attain a better environment [20]. Plants communicate with microorganisms via cell growth phases, where the roots contribute to the rhizospheric environment via the lysis of root cells, polysaccharides, and volatile organic carbon that activates the symbiotic interaction with PGPMs [21,22]. Thus, plants communicate with microorganisms during their growth period through their root exudates and secretion of the various signaling molecules that stimulate and promote the survival of the microorganisms, increasing the degradation and transformation of organic pollutants [23]. 

Among the ubiquitous microorganisms in nature, there are plant growth-promoting microorganisms (PGPMs). These PGPMs are a specialized group of microorganisms associated with the roots that promote the growth of plants and protect plants from abiotic stress and pathogens. Several studies have observed that PGPMs enhance the metabolic response in plants in a species-specific manner [4,24,25]. The soil microorganisms of plant roots are divided into three sections—the rhizosphere, rhizoplane, and endosphere [21,26]—which usually influence an area of several centimeters around the plant’s roots with density ranges from 10^8^ to 10^11^ colony-forming units (CFUs) per gram of root [26]. A study conducted on samples of the rhizosphere of the Khalas date palm showed that the majority of the identified sequences (86%) belonged to bacteria [6,7,8]. Another study analyzed the rhizosphere of two cultivars of the Khalas date palm and found that the rhizosphere of the Sukkari and Khalas cultivars possessed 62% and 86% bacteria, respectively [6,7,8]. Microbes stimulate and protect plants, while plants in turn provide nutrients to microbes; this relationship is not only for survival purposes, but also for prominent benefits such as phytoremediation. The biomass and nutrient uptake of Sorghum plants increased after inoculation with PGPMs alone or in combination with mycorrhiza in a soil-based medium [27]. Additionally, *Banana Berangan* seedlings showed an increase in chlorophyll content, biomass, and the growth of shoots and roots following inoculation with *Bacillus sphaericus* and *Azospirillum* sp. [28,29]. Some studies have applied PGPMs to seeds or seedlings or used microbial siderophores in the hydroponics medium. For instance, the application of *Gluconacetobacter diazotrophicus* and *Azospirillum brasilense* siderophores in hydroponic mediums increased the nutritional value of strawberries by increasing the iron content [30]. Furthermore, the application of PGPMs (*Calothrix* sp., *Anabaena cylindrica*, *Chryseobacterium balustinum*, *Pseudomonas simiae*, and *Pseudomonas fluorescens*) to seedlings increased the phytohormones and growth indicators in the shoots and roots of *Triticum aestivum* after 17 days of treatment [31]. 

## 3. The Role of PGPMs in Plant Detoxification

The interaction of plants with microorganisms augments the process of phytoremediation, as well as paves the way for multiple cleaning options, such as multiple elemental remediation [31,32]. Plants interact with microbes to survive in hostile environments such as saline or heavy metal-enriched soil [33]. The resistance properties of the plants or the stimulating activity of the microbial rhizosphere develop and emerge during the growth of the plants in the contaminated regions [34]. The insertion of specific strains of microbes in seeds has been performed to colonize the roots so that the microbes remain on the root systems [35]. Microbes can affect the process of phytoremediation via multiple mechanisms. Microbial-mediated degradation of organic pollutants and the uptake of heavy metals occurs through bioaugmentation and biostimulation. Biostimulation is defined as the stimulation of microorganisms residing in the contaminated soil by incorporating nutrients such as N and P, as well as electron donors for the degradation of harmful compounds [27,29]. Diverse molecules secreted by plants owing to microbe interactions serve as chelating agents that increase the phytoavailability of the organic pollutants; hence, such microbes play a vital role in the phytoremediation process [33]. The utilization of plants for the management of soil contaminated with radionuclides (waste from nuclear power reactors, sewage sludge, and waste from power plants) and heavy metals is a more suitable modality for bioremediation, and the interaction between plants [36] and microbes ensures the more effective remediation of organic pollutants [37]. The addition of genetically engineered or natural microbes into contaminated soil to degrade toxic compounds is known as bioaugmentation [29]. The combination of *Brassica napus* and bioaugmentation by actinobacteria is more effective in transforming hexavalent chromium into trivalent chromium after the addition of organic matter into the soil [38]. Monti et al. [39] demonstrated that *Pseudomonas fluorescens*-mediated bioaugmentation is able to degrade 2,4-DNT (dinitrotoluene), thus decreasing the toxicity for *Arabidopsis thaliana*. Additional studies have shown that composite soil, consisting mainly of active microorganisms and organic matter, acts as a biostimulator and degrades organic pollutants (OPs) of special concern, including polycyclic aromatic hydrocarbons (PAHs), pesticides, and petroleum [40]. Poor soil rhizospheres enriched with various kinds of microbial activity were shown to enhance the availability of elements, especially base elements [2,3,4]. Fungi are also beneficial for the process of phytoremediation either in direct or indirect interactions with pollutants. Fungi reside between the air–water interface and phytoremediation mediated by fungi has benefits over bacteria as they need a water phase for their activity [41]. Other studies have observed that mycorrhizal fungi affect the bioavailability of metals via alteration of the biochemical properties of the soil and the components of root exudates [42,43]. Arbuscular mycorrhizal fungi (AMF) can enhance the surface area for absorption in plant roots [44] resulting in enhanced metal, nutrient, and water uptake. Dhawi et al. [45] found that inoculation with both endomycorrhiza and plant growth-promoting bacteria (PGPB) enhanced plant biomass as well as the yield of sugar in foxtail millet. 

Vamerali et al. [46] found that phytohormones released by AMF can encourage phytoremediation as well as promote the growth of plants. 

A study conducted on Zn-contaminated soil showed that the addition of AMF enhanced the productivity of *Trifolium pratense* (red clover plant) and the accumulation of Zn in the roots [47]. In addition to the role of fungi in the removal of heavy metals, they also help in the removal of organic pollutants such as atrazine, 2,4-dichlorophenol, polychlorinated biphenyls (PCBs), 2,4,6-trinitrotoluene, and polycyclic aromatic hydrocarbons (PAHs), which can be removed by ectomycorrhizal fungi (ECM) [48]. Several microbes possess the remarkable ability to transform organic pollutants into less toxic compounds; these include *Pseudomonas aeruginosa, Cupriavidus metallidurans*, *Pseudomonas putida*, *Aspergillus fumigatus*, *Aspergillus versicolor*, *Aspergillus tereus*, *Candida utilis*, *Penicillum chrysogenum*, *Saccharomyces cerevisiae*, *Rhodotorula mucilaginosa*, and *Phanerochaete chrysosporium* [49]. 

Numerous previous studies have shown that PGPMs increase the solubility of metals by releasing protons and organic anions [50]. Another study revealed that PGPB enhance the root biomass and uptake of elements in *Sorghum* plants either alone or in combination with mycorrhiza [2,51]. PGPMs are able to enhance the efficacy of the process of phytoremediation by increasing the tolerance of plants to metals and pathogens, releasing siderophores and enhancing the biomass, the growth of plants, and the uptake and translocation of heavy metals [25]. PGPMs promote the growth of plants through the secretion of phytohormones such as ethylene, cytokinins, and gibberellic, abscisic, salicylic, and jasmonic acids [52,53,54]. PGPMs also affect the post-embryonic development of roots [55]. 

Braud et al. [56] observed that the inoculation of maize plants with bacteria that produced siderophores increased the bioavailability and uptake of Pb and Cr, thus enhancing the ability for phytoremediation in the maize. PGPMs enhance the growth of plants by reducing the ethylene production through secretion of ACC (1-aminocyclopropane-1-carboxylic acid) deaminase enzymes [57]. Previous studies have reported that *Pseudomonas*, *Serratia*, and *Bacillus* enhance the growth of plants via secretion of ACC deaminase enzymes [58,59,60]. Additionally, PGPMs promote the growth of lateral roots and root hairs through the secretion of bacterial auxin [61], enhancing the process of phytoremediation. The most prevalent microbes employed for increasing the growth of plants and physiological activities include *Enterobacter*, *Pseudomonas*, *Arthrobacter*, *Flavobacterium*, *Beijerinkia*, *Glucanoacetobacterium*, *Erwinia*, *Klebsiella*, *Serratia*, and *Bacillus* [62].

Xerophile microorganisms such as *Bacillus*, with 27 strains isolated from rhizospheric soils in Tunisia, showed inhibitory potentials against Gram-positive and Gram-negative test bacteria [63]. In another study, 116 cultured bacteria isolated from the rhizospheres and endospheres of four native desert plants, *Tribulus terrestris*, *Zygophyllum simplex*, *Panicum turgidum*, and *Euphorbia granulata*, showed biochemical properties related to nutrient acquisition, hormone production, and salinity tolerance [64].

## 4. The Role of PGPMs in Abiotic Stress Tolerance

Abiotic stress factors such as extreme temperature, drought, and hypersalinity have a negative impact on plant growth that causes the reduced production of crops worldwide. Hypersalinity and drought are the prevalent causes of low crop yields [65]. Plant and microbe interactions are useful to circumvent hostile conditions such as drought, hypersalinity, and extreme temperature in abiotically stressed plants. Phytohormones play a crucial role in modulating the morphology of roots. Several studies have reported that the microbes associated with plants have the capability to modulate the growth of roots [49]. ACC deaminase activity is a characteristic feature of both rhizospheric and endophytic bacteria, and is useful for plants experiencing abiotic stress conditions [66]. The ACC deaminase activity of microbes associated with plants uses the precursor ethylene as a source of nitrogen, thus reducing the ethylene level and resulting in the promotion of root growth [67]. Proline is another substance formed by plants that plays an important role in the stabilization of free-radical scavenging, the adjustment of osmosis, and sub-cellular structures. PGPMs play an essential role in the stress tolerance associated with proline induction. Rai et al. [68] and Ait Barka et al. [69] observed that the synthesis of proline is enhanced in plants after inoculation with *Burkholderia* bacteria.

### 4.1. Tolerance to a Drought Environment

Drought has a multi-dimensional stress impact that affects the biochemical, physiological, morphological, and molecular characteristics of plants, and lowers the productivity and growth of plants, resulting in the loss of crop yields. It has been reported to reduce national cereal production by 9–10% [70]. Microbes associated with plants mitigate the effects of drought by enhancing water circulation in the plant, secreting exopolysaccharides, and stimulating resistance genes and the synthesis of proline, indole acetic acid (IAA), and aminocyclopropane-1-carboxylic (ACC) deaminase [71]. Different plants tolerate drought conditions through different mechanisms, which are associated with different microorganisms such as endophytic fungi, mycorrhizal fungi, and PGPMs [68]. In drought environments, specific adaptations of roots have shown an increase in various roots having smaller diameters and deeper root systems [72]. Shoot growth is normally inhibited in drought conditions [73]; however, Vardharajula et al. [74] revealed that shoot growth increased in drought conditions after inoculation with Bacillus species. Osmotic adjustment is another mechanism through which plants tolerate drought conditions [75]. Proline is an important osmolyte that accumulates in plants during drought conditions [75]. The expression of several enzymes including SOD (superoxide dismutase), CAT (catalase), GR (glutathione reductase), POX (peroxidase), and APX (ascorbate peroxidase) was found to increase under drought conditions as the drought induced the formation of reactive oxygen species (ROS) in plants. An increase in scavenging systems during drought conditions has been associated with certain enzymes in plants [75]. PGPMs might respond to stress by regulating phytohormones and antioxidants, and increase plant survival by triggering several genes associated with growth and development. In a previous study, sorghum roots planted in heavy metal-impacted soil inoculated with PGPMs showed the increased expression of two protein groups. The first group, including Sulfatase, FGGY_C, and Phosphodiesterase, was associated with DNA regulation. The second group was associated with stress tolerance proteins such as HSP70. At the same time, sorghum roots expressed bacterial transcriptional regulators, thus indicating a phytomicrobial-mutable interaction and benefit [22].

### 4.2. Tolerance to Extreme Temperatures

The majority of biological reactions depend upon an optimal temperature. Hence, an alteration in temperature (either too cold or too hot) affects biological reactions, culminating in alterations in the physiological, biochemical, and morphological traits of plants. Grover et al. [71] reported that several bacteria help plants to tolerate extreme temperatures. One study showed that *Cuvularia* species (endophytic fungi) were thermo-tolerant to high temperatures from 50–65 °C [76]; however, the plants were not able to tolerate a temperature of more than 38 °C when the fungi and plants grew separately. Another study reported that the NBRI0987 strain of *Pseudomonas* species increased plant biomass under a high temperature. In the same study, bacteria mediated the tolerance to colder temperatures, which was significantly correlated with induced systemic resistance (ISR) [68].

### 4.3. Tolerance to a Hypersaline Environment

Hypersalinity has detrimental effects on agriculture as it affects plant productivity and plant growth. Bui et al. [77] showed that salinity affects about 6% of the global land mass, especially in semi-arid and arid regions. A study conducted by Bashan et al. [78] found that PGPMs improve the capacity of plants to tolerate saline conditions. The development of seeds under saline conditions can be supported by exopolysaccharides produced by plants associated with bacteria, as well as by nitrogen-fixing bacteria [79]. Bacteria promote the germination of seeds and support plant growth through the secretion of phytohormones, especially IAA and GA [80]. Bacteria also increase the flow of potassium ions from the roots to the shoots to ameliorate the effects of sodium toxicity under saline conditions [68]. Grover et al. [71] showed that the development and growth of lettuce, peppers, tomatoes, and beans grown in saline conditions are supported by PGPMs. Another study reported the increased secretion of exopolysaccharides in wheat seedlings that stimulated plant growth and restricted the uptake of sodium ions after inoculation with bacteria [81]. Sen et al. [82] showed that the Pseudomonas species increase root colonization during rice germination by secreting exopolysaccharides. 

## 5. Possibilities of Using PGPMs in Hydroponics and Vertical Farming

Most modern hydroponic systems are categorized into seven main types (wicking systems, ebb and flow (flood and drain), drip irrigation, nutrient film technique (NFT), deep water cultures, aeroponics, and aquaponics) based on the application of nutrient solutions and the plant root system [83]. While a soil system supports the plants’ growth environment via PGPM colonization, and other lower-order animals, such as earthworms, that help in nutrient cycling [84], a hydroponic system provides a controlled and optimum environment to extract higher antioxidant contents in comparison to a soil-based system [85]. Vertical farming systems are compiled by stacking traditional hydroponic, aqua-ponic, or aeroponic horizontal layers one over the other to achieve a vertical structure [86]. The vertical farming system can be controlled in a greenhouse or by an installed system. There are many challenges in vertical farming systems or hydroponics. Pathogens can easily spread throughout an entire crop due to the proximity of the plants and the re-circulated nutrient solution [87]. Another challenge is the addition of nutrients. Some challenges that might be mitigated using PGPMs are pathogen control and nutrition enhancement [28,88,89]. However, various studies have shown that the inoculation of plants with a bacterial consortium has a stronger impact on plant growth, while also helping to reduce abiotic and biotic stress [4,5,6,90]. For example, the use of Paenibacillus polymyxa and Bacillus megaterium in combination with Rhizobium was shown to increase the biomass of Phaseolus vulgaris plants compared to inoculation with Rhizobium alone [91]. Moreover, legume inoculation with Rhizobium and Pseudomonas improved the concentration of N, K, and Na, as well as biomass and yield [92]. The nodulation, nitrogen fixation, and nutrient uptake increased in Glycine max post-inoculation with a consortium of *Bradyrhizobium* and *Streptomyces griseoflavus* [93]. The ability of hydroponics or vertical farming to include PGPMs was evident in the study by Wiggins et al. [94], where the use of several substrates with a reduced amount of fertilizer showed promising results in the productivity of lettuce varieties. Hydroponics or vertical farming PGPMs should follow the selection criteria reported by Vejan et al. [95], e.g., effective root system colonization, stability under changes in environmental conditions, and high competitiveness with substrate microorganisms to address issues associated with soil-free systems. PGPMs can reduce salinity stress [96], which will reduce the cost of water treatment and subsequent system sterilization [97,98]. Selecting anti-pathogen microorganisms might boost plant resistance [63]. Thus, the introduction of PGPMs to vertical or hydroponics systems might take several forms, e.g., inoculating seeds or seedlings prior to sowing [99,100] or adding siderophores or microbial osmolytes to nutrient solutions or substrates [101]. Physical sterilization methods also need to be considered, such as UV light in the case of adding PGPM substrates to hydroponic systems [102] to avoid contamination. Several cases have been reported using PGPMs in a soil-free medium, as shown in Table 1. These studies show that seedling inoculation with a microbial suspension increased plant growth, and consequently, the total biomass in plants such as *Banana Berangan*, *Triticum aestivum*, tomato, and *Glycine max* (L.) Merr. [28,31,103,104]. In addition, using biostimulant extracts or siderophores added to the medium in a soil-free system had a positive impact on plants such as lettuce and strawberries [30,105]. Therefore, defining the mode of PGPM application according to different soil-free systems is crucial to maximizing their positive impact. Moreover, the use of PGPM in a soil-free system can improve recycled water quality by breaking down organic matter, reducing the buildup of harmful substances, and helping to balance the pH levels. This results in a more efficient use of recycled water, as the water can be reused multiple times without the need for frequent changes or replacements. In addition, PGPM can also help to mitigate the negative effects of water-borne pathogens that may be present in recycled water. This is particularly important in closed-loop systems where water is recirculated multiple times. The presence of PGPM can help to maintain water quality and ensure the health of the plants. Overall, the use of PGPM in a soil-free system can provide numerous benefits and make vertical farming or hydroponic systems more sustainable and efficient methods of agriculture. In Table 2, we suggest the PGPM application mode according to the susceptibility of the plant root system to pathogens and increased humidity. In addition, pre-treatment or inoculation of the seeds or seedlings with a microbial suspension is recommended for all soil-free systems, except drip-irrigation systems. 

## 6. Conclusions

Soil-free systems such as hydroponics and vertical farming support plant growth by providing a controlled and optimum environment to extract higher antioxidant contents in comparison to soil-based systems. In soil-free systems, such as vertical farming and hydroponics, the challenges related to nutrient availability might be resolved by PGPMs. Therefore, we propose that using PGPMs in aeroponics and aquaponics systems before transferring seeds or seedlings to the soil-free system might increase plant growth by improving the production of plant hormones and the utilization of elements. However, we suggest the use of a PGPM bio-stimulant extract in hydroponic systems. 

## Figures and Tables

**Table 1:** Previous studies on plant growth-promoting microorganisms’ (PGPMs) mode of application in a soil-free system, plant types used, and their influence.

Plant	Mode of PGPM Application	Type of PGPM	Influence	References
Banana Berangan’ (*Musa* spp. dessert type)	Seedling inoculation with microbial suspension	*Bacillus sphaericus* and *azospirillum*	Increase in root formation, leaf area, chlorophyll content, and consequently, total biomass	[28]
Strawberries	Siderophores added to hydroponic medium	*Gluconacetobacter diazotrophicus* and *azospirillum brasilense*	Increased the nutrition of iron	[30]
Triticum aestivum	Seedling inoculation with microbial suspension	*Calothrix sp., anabaena cylindrica, chryseobacterium balustinum, pseudomonas simiae,* and *pseudomonas fluorescen*	Increased the growth, plant height, dry shoot mass, total nutrients, and the ability to produce indole acetic acid	[31]
Lettuce	Biostimulant extract added to hydroponic medium	*Bacillus* spp.	Minimized salt stress	[96]
Tomato	Seedling inoculation with microbial suspension	*Penicillium brevicompactum, penicillium solitum strain 1, pseudomonas fluorescens subgroup g strain 2, pseudomonas marginalis, pseudomonas putida subgroup b strain 1, pseudomonas syringae strain 1,* and *trichoderma atroviride*	Plant growth and development in the absence of pathogens (antagonistic activity against *Pythium ultimum*)	[103]
*Glycine max (L.) Merr.*	Seedling inoculation with microbial suspension	Bacteria, yeasts, *mycorrhiza*, and *Trichoderma*	Higher density of smaller stomata, thicker palisade parenchyma, larger intercellular spaces in the mesophyll; increased photosynthetic traits, growth and seed production	[104]
Lettuce (*Salanova^®^ Lactuca sativa* and *Salanova^®^ Red Crisp*).	Bio-stimulant extract added to hydroponic medium	Phycocyanin-rich spirulina extract	Reduced time from seed to harvest by 6 days, increased yield by 12.5%, and improved antioxidant flavonoid levels	[105]

**Table 2:** Types of hydroponic systems and suggested mode of PGPM application.

Hydroponic Types	Definition	Suggested Mode of PGPM Application
Wicking System	Small-scale production plants (small plants, e.g., herbs and leafy greens)	Pre-treatment seedling inoculation with microbial suspension
Ebb and Flow (Flood and Drain)	Seedling cultivation in commercial settings	Pre-treatment seedling inoculation with microbial suspension
Drip Irrigation	Commercial production for larger fruiting crops such as tomatoes, cucumbers, peppers, and strawberries	Synchronize treatment: Bio-stimulant extract added to hydroponic medium
Nutrient Film Technique	In commercial use for smaller leafy crops such as lettuce	Pre-treatment seedling inoculation with microbial suspension
Deep Water Culture	In commercial settings deep water culture systems are used for small leafy plants such as lettuce or herbs	Pre-treatment seedling inoculation with microbial suspension
Aeroponics	Commercial growers to produce small leafy plants and potato mini-tubers	Pre-treatment seedling inoculation with microbial suspension
Aquaponics	Growing fish and plants in the same system	Pre-treatment seedling inoculation with microbial suspension

## References

[1] Ma Y. (2019). Biotechnological potential of plant-microbe interactions in environmental decontamination. Front. Plant Sci..

[2] Dhawi F., Datta R., Ramakrishna W. (2016). Mycorrhiza and heavy metal resistant bacteria enhance growth, nutrient uptake and alter metabolic profile of sorghum grown in marginal soil. Chemosphere.

[3] Dhawi F., Datta R., Ramakrishna W. (2017). Proteomics provides insights into biological pathways altered by plant growth promoting bacteria and arbuscular mycorrhiza in sorghum grown in marginal soil. Biochim. Biophys. Acta Proteins Proteom..

[4] Dhawi F., Hess A. (2017). Plant growth-prompting bacteria influenced metabolites of *Zea mays* var. amylacea and *Pennisetum americanum* p. in a species-specific manner. Adv. Biol. Chem..

[5] Dhawi F., Hess A. (2017). Poor-soil rhizosphere enriched with different microbial activities influence the availability of base elements. Open J. Ecol..

[6] Dhawi F., Alsanie S.I. (2021). So it is above, so it is below: Microbial Pathways Associated with Date Palm Trees. Ann. Rom. Soc. Cell Biol..

[7] Dhawi F. (2022). Investigating Two Date Palm Cultivars Microbial Pathways. J. Hunan Univ. Nat. Sci..

[8] Dhawi F., Alsanie S.I. (2022). Investigation of Microbial Community Structure and Diversity in the Rhizosphere of Date Palm (*Phoenix dactylifera* L.), Sukkari Cultivar. J. Hunan Univ. Nat. Sci..

[9] Mehmood T., Gaurav G.K., Cheng L., Klemeš J.J., Usman M., Bokhari A., Lu J. (2021). A review on plant-microbial interactions, functions, mechanisms and emerging trends in bioretention system to improve multi-contaminated stormwater treatment. J. Environ. Manag..

[10] Conlon R., Wang M., Germaine X.L., Mali R., Dowling D., Germaine K.J. (2022). Ecopiling: Beneficial Soil Bacteria, Plants, and Optimized Soil Conditions for Enhanced Remediation of Hydrocarbon Polluted Soil. Good Microbes Med. Food Prod. Biotechnol. Bioremediat. Agric..

[11] Augusta A.C., Bertha E.E., Eromosele A.S. (2022). Plant-Microbe Interaction: Prospects and Applications in Sustainable Environmental Management. Plant Hormones: Recent Advances. New Perspectives and Applications.

[12] Schnitzler W.H. (2013). Urban hydroponics–facts and vision. SEAVEG 2012 High Value Vegetables in Southeast Asia: Production, Supply and Demand.

[13] Despommier D. (2013). Farming up the city: The rise of urban vertical farms. Trends Biotechnol..

[14] Kaur G., Chawla P. (2021). All about Vertical Farming: A Review. Turk. J. Comput. Math. Educ..

[15] Kulak M., Graves A., Chatterton J. (2013). Reducing greenhouse gas emissions with urban agriculture: A Life Cycle Assessment perspective. Landsc. Urban Plan.

[16] Koriesh E.M., Abo-Soud I.H. (2020). Facing Climate Change: Urban Gardening and Sustainable Agriculture. Climate Change Impacts on Agriculture and Food Security in Egypt.

[17] Rajan P., Lada R.R., MacDonald M.T. (2019). Advancement in indoor vertical farming for microgreen production. Am. J. Plant Sci..

[18] Singh B.K., Liu H., Trivedi P. (2020). Eco-holobiont: A new concept to identify drivers of host-associated microorganisms. Environ. Microbiol..

[19] Zilber-Rosenberg I., Rosenberg E. (2008). Role of microorganisms in the evolution of animals and plants: The hologenome theory of evolution. FEMS Microbiol. Rev..

[20] Kothe E., Turnau K. (2018). Mycorrhizosphere communication: Mycorrhizal fungi and endophytic fungus-plant interactions. Front. Microbiol..

[21] de la Fuente Cantó C., Simonin M., King E., Moulin L., Bennett M.J., Castrillo G., Laplaze L. (2020). An extended root phenotype: The rhizosphere, its formation and impacts on plant fitness. Plant J..

[22] Dhawi F. (2020). Plant growth promoting Rhizobacteria (PGPR) regulated Phyto and microbial beneficial protein interactions. Open Life Sci..

[23] Ghitti E., Rolli E., Crotti E., Borin S. (2022). Flavonoids Are Intra-and Inter-Kingdom Modulator Signals. Microorganisms.

[24] Pratush A., Kumar A., Hu Z. (2018). Adverse effect of heavy metals (As, Pb, Hg, and Cr) on health and their bioremediation strategies: A review. Int. Microbiol..

[25] Ma Y., Dias M.C., Freitas H. (2020). Drought and salinity stress responses and microbe-induced tolerance in plants. Front. Plant Sci..

[26] Hacquard S., Garrido-Oter R., González A., Spaepen S., Ackermann G., Lebeis S., McHardy A.C., Dangl J.L., Knight R., Ley R. (2015). Microbiota and host nutrition across plant and animal kingdoms. Cell Host Microbe.

[27] Ojuederie O.B., Babalola O.O. (2017). Microbial and plant-assisted bioremediation of heavy metal polluted environments: A review. Int. J. Environ. Res. Public Health.

[28] Baset M.M., Shamsuddin Z.H., Wahab Z., Marziah M. (2010). Effect of plant growth promoting rhizobacterial (PGPR) inoculation on growth and nitrogen incorporation of tissue-cultured'musa'plantlets under nitrogen-free hydroponics condition. Aust. J. Crop Sci..

[29] Goswami M., Chakraborty P., Mukherjee K., Mitra G., Bhattacharyya P., Dey S., Tribedi P. (2018). Bioaugmentation and biostimulation: A potential strategy for environmental remediation. J. Microbiol. Exp..

[30] Delaporte-Quintana P., Lovaisa N.C., Rapisarda V.A., Pedraza R.O. (2020). The plant growth promoting bacteria *Gluconacetobacter diazotrophicus* and *Azospirillum brasilense* contribute to the iron nutrition of strawberry plants through siderophores production. Plant Growth Regul..

[31] Phieler R., Merten D., Roth M., Büchel G., Kothe E. (2015). Phytoremediation using microbially mediated metal accumulation in *Sorghum bicolor*. Environ. Sci. Pollut. Res..

[32] Basu S., Rabara R.C., Negi S., Shukla P. (2018). Engineering PGPMOs through gene editing and systems biology: A solution for phytoremediation?. Trends Biotechnol..

[33] Abhilash P.C., Powell J.R., Singh H.B., Singh B.K. (2012). Plant–microbe interactions: Novel applications for exploitation in multipurpose remediation technologies. Trends Biotechnol..

[34] Kuiper I., Lagendijk E.L., Bloemberg G.V., Lugtenberg B.J. (2004). Rhizoremediation: A beneficial plant-microbe interaction. Mol. Plant-Microbe Interact..

[35] Mendis H.C., Thomas V.P., Schwientek P., Salamzade R., Chien J.T., Waidyarathne P., Kloepper J., De La Fuente L. (2018). Strain-specific quantification of root colonization by plant growth promoting rhizobacteria *Bacillus firmus* I-1582 and *Bacillus amyloliquefaciens* QST713 in non-sterile soil and field conditions. PLoS ONE.

[36] Jain S., Arnepalli D.N. (2019). Biominerlisation as a remediation technique: A critical review. Geotechnical Characterisation and Geoenvironmental Engineering.

[37] Chibuike G.U., Obiora S.C. (2014). Heavy metal polluted soils: Effect on plants and bioremediation methods. Appl. Environ. Soil Sci..

[38] Lacalle R.G., Aparicio J.D., Artetxe U., Urionabarrenetxea E., Polti M.A., Soto M., Garbisu C., Becerril J.M. (2020). Gentle remediation options for soil with mixed chromium (VI) and lindane pollution: Biostimulation, bioaugmentation, phytoremediation and vermiremediation. Heliyon.

[39] Monti M.R., Smania A.M., Fabro G., Alvarez M.E., Argarana C.E. (2005). Engineering Pseudomonas fluorescens for biodegradation of 2,4-dinitrotoluene. Appl. Environ. Microbiol..

[40] Ren X., Zeng G., Tang L., Wang J., Wan J., Wang J., Deng Y., Liu Y., Peng B. (2018). The potential impact on the biodegradation of organic pollutants from composting technology for soil remediation. Waste Manag..

[41] Kumar A., Chaturvedi A.K., Yadav K., Arunkumar K.P., Malyan S.K., Raja P., Kumar R., Khan S.A., Yadav K.K., Rana K.L. (2019). Fungal phytoremediation of heavy metal-contaminated resources: Current scenario and future prospects. Recent Advancement in White Biotechnology through Fungi.

[42] Bhantana P., Rana M.S., Sun X.C., Moussa M.G., Saleem M.H., Syaifudin M., Shah A., Poudel A., Pun A.B., Bhat M.A. (2021). Arbuscular mycorrhizal fungi and its major role in plant growth, zinc nutrition, phosphorous regulation and phytoremediation. Symbiosis.

[43] Sarwar N., Imran M., Shaheen M.R., Ishaque W., Kamran M.A., Matloob A., Rehim A., Hussain S. (2017). Phytoremediation strategies for soils contaminated with heavy metals: Modifications and future perspectives. Chemosphere.

[44] Göhre V., Paszkowski U. (2006). Contribution of the arbuscular mycorrhizal symbiosis to heavy metal phytoremediation. Planta.

[45] Dhawi F., Datta R., Ramakrishna W. (2018). Metabolomics, biomass and lignocellulosic total sugars analysis in foxtail millet (*Setaria italica*) inoculated with different combinations of plant growth promoting bacteria and mycorrhiza. Commun. Plant Sci..

[46] Vamerali T., Bandiera M., Mosca G. (2010). Field crops for phytoremediation of metal-contaminated land. A review. Environ. Chem. Lett..

[47] Chen B.D., Li X.L., Tao H.Q., Christie P., Wong M.H. (2003). The role of arbuscular mycorrhiza in zinc uptake by red clover growing in a calcareous soil spiked with various quantities of zinc. Chemosphere.

[48] Joner E.J., Leyval C. (2009). Phytoremediation of organic pollutants using mycorrhizal plants: A new aspect of rhizosphere interactions. Sustainable Agriculture.

[49] Arantza S.J., Hiram M.R., Erika K., Chávez-Avilés M.N., Valiente-Banuet J.I., Fierros-Romero G. (2022). Bio- and phytoremediation: Plants and microbes to the rescue of heavy metal polluted soils. SN Appl. Sci..

[50] Becerra-Castro C., Prieto-Fernández A., Álvarez-Lopez V., Monterroso C., Cabello-Conejo M.I., Acea M.J., Kidd P.S. (2011). Nickel solubilizing capacity and characterization of rhizobacteria isolated from hyperaccumulating and non-hyperaccumulating subspecies of *Alyssum serpyllifolium*. Int. J. Phytoremediat..

[51] Gill R.A., Ahmar S., Ali B., Saleem M.H., Khan M.U., Zhou W., Liu S. (2021). The role of membrane transporters in plant growth and development, and abiotic stress tolerance. Int. J. Mol. Sci..

[52] Malamy J.E. (2005). Intrinsic and environmental response pathways that regulate root system architecture. Plant Cell Environ..

[53] Doornbos R.F., Geraats B.P., Kuramae E.E., Van Loon L.C., Bakker P.A. (2011). Effects of jasmonic acid, ethylene, and salicylic acid signaling on the rhizosphere bacterial community of *Arabidopsis thaliana*. Mol. Plant-Microbe Interact..

[54] Osmont K.S., Sibout R., Hardtke C.S. (2007). Hidden branches: Developments in root system architecture. Annu. Rev. Plant Biol..

[55] Verbon E.H., Liberman L.M. (2016). Beneficial microbes affect endogenous mechanisms controlling root development. Trends Plant science.

[56] Braud A., Jézéquel K., Bazot S., Lebeau T. (2009). Enhanced phytoextraction of an agricultural Cr-and Pb-contaminated soil by bioaugmentation with siderophore-producing bacteria. Chemosphere.

[57] Chang P., Gerhardt K.E., Huang X.D., Yu X.M., Glick B.R., Gerwing P.D., Greenberg B.M. (2014). Plant growth-promoting bacteria facilitate the growth of barley and oats in salt-impacted soil: Implications for phytoremediation of saline soils. Int. J. Phytoremediation.

[58] Babu A.G., Kim J.D., Oh B.T. (2013). Enhancement of heavy metal phytoremediation by *Alnus firma* with endophytic *Bacillus thuringiensis* GDB-1. J. Hazard. Mater..

[59] Agnello A.C., Bagard M., van Hullebusch E.D., Esposito G., Huguenot D. (2016). Comparative bioremediation of heavy metals and petroleum hydrocarbons co-contaminated soil by natural attenuation, phytoremediation, bioaugmentation and bioaugmentation-assisted phytoremediation. Sci. Total Environ..

[60] Dong R., Gu L., Guo C., Xun F., Liu J. (2014). Effect of PGPR Serratia marcescens BC-3 and AMF Glomus intraradices on phytoremediation of petroleum contaminated soil. Ecotoxicology.

[61] DalCorso G., Fasani E., Manara A., Visioli G., Furini A. (2019). Heavy metal pollutions: State of the art and innovation in phytoremediation. Int. J. Mol. Sci..

[62] Ma Y., Oliveira R.S., Freitas H., Zhang C. (2016). Biochemical and molecular mechanisms of plant-microbe-metal interactions: Relevance for phytoremediation. Front. Plant Sci..

[63] Nasfi Z., Busch H., Kehraus S., Linares-Otoya L., König G.M., Schäberle T.F., Bachoual R. (2018). Soil bacteria isolated from tunisian arid areas show promising antimicrobial activities against gram-negatives. Front. Microbiol..

[64] Eida A.A., Ziegler M., Lafi F.F., Michell C.T., Voolstra C.R., Hirt H., Saad M.M. (2018). Desert plant bacteria reveal host influence and beneficial plant growth properties. PLoS ONE.

[65] He M., He C.Q., Ding N.Z. (2018). Abiotic stresses: General defenses of land plants and chances for engineering multistress tolerance. Front. Plant Sci..

[66] Saleem M., Arshad M., Hussain S., Bhatti A.S. (2007). Perspective of plant growth promoting rhizobacteria (PGPR) containing ACC deaminase in stress agriculture. J. Ind. Microbiol. Biotechnol..

[67] Glick B.R., Penrose D.M., Li J. (1998). A model for the lowering of plant ethylene concentrations by plant growth-promoting bacteria. J. Theor. Biol..

[68] Rai A., Borpatragohain B., Sahoo S. (2019). Role of plant-microbe interactions on abiotic stress tolerance in plants: A review. Int. J. Agric. Plant Sci..

[69] Ait Barka E., Nowak J., Clément C. (2006). Enhancement of chilling resistance of inoculated grapevine plantlets with a plant growth-promoting rhizobacterium, *Burkholderia phytofirmans* strain PsJN. Appl. Environ. Microbiol..

[70] Lesk C., Rowhani P., Ramankutty N. (2016). Influence of extreme weather disasters on global crop production. Nature.

[71] Grover M., Farrugia G., Lurken M.S., Bernard C.E., Faussone–Pellegrini M.S., Smyrk T.C., Parkman H.P., Abell T.L., Snape W.J., Hasler W.L. (2011). Cellular changes in diabetic and idiopathic gastroparesis. Gastroenterology.

[72] Comas L.H., Becker S.R., Cruz V.M., Byrne P.F., Dierig D.A. (2013). Root traits contributing to plant productivity under drought. Front. Plant Sci..

[73] Seleiman M.F., Al-Suhaibani N., Ali N., Akmal M., Alotaibi M., Refay Y., Dindaroglu T., Abdul-Wajid H.H., Battaglia M.L. (2021). Drought stress impacts on plants and different approaches to alleviate its adverse effects. Plants.

[74] Vardharajula S., Zulfikar Ali S., Grover M., Reddy G., Bandi V. (2011). Drought-tolerant plant growth promoting *Bacillus* spp.: Effect on growth, osmolytes, and antioxidant status of maize under drought stress. J. Plant Interact..

[75] Abobatta W.F. (2019). Drought adaptive mechanisms of plants—A review. Adv. Agric. Environ. Sci..

[76] Redman R.S., Sheehan K.B., Stout R.G., Rodriguez R.J., Henson J.M. (2002). Thermotolerance generated by plant/fungal symbiosis. Science.

[77] Bui L., Luo H., Gunther W.R., Román-Leshkov Y. (2013). Domino reaction catalyzed by zeolites with Brønsted and Lewis acid sites for the production of γ-valerolactone from furfural. Angew. Chem..

[78] Bashan Y., de-Bashan L.E., Prabhu S.R., Hernandez J.P. (2014). Advances in plant growth-promoting bacterial inoculant technology: Formulations and practical perspectives (1998–2013). Plant Soil.

[79] Wu J. (2012). Advances in K-Means Clustering: A Data Mining Thinking.

[80] Egamberdieva D., Wirth S.J., Alqarawi A.A., Abd_Allah E.F., Hashem A. (2017). Phytohormones and beneficial microbes: Essential components for plants to balance stress and fitness. Front. Microbiol..

[81] Grover P., Sahai A. Shannon meets Tesla: Wireless information and power transfer. Proceedings of the 2010 IEEE International Symposium on Information Theory.

[82] Sen S., Chandrasekhar C.N. (2014). Effect of PGPR on growth promotion of rice (*Oryza sativa* L.) under salt stress. Asian J. Plant Sci. Res..

[83] Stegelmeier A.A., Rose D.M., Joris B.R., Glick B.R. (2022). The Use of PGPB to Promote Plant Hydroponic Growth. Plants.

[84] Barker K.R., Koenning S.R. (1998). Developing sustainable systems for nematode management. Annu. Rev. Phytopathol..

[85] Zapata-Vahos I.C., Rojas-Rodas F., David D., Gutierrez-Monsalve J.A., Castro-Restrepo D. (2020). Comparison of antioxidant contents of green and red leaf lettuce cultivated in hydroponic systems in greenhouses and conventional soil cultivation. Rev. Fac. Nac. Agron. Medellín.

[86] Nair A.G., Chacko A., Mohan G., Francis T.K. (2015). Smart vertical farming using hydroponics. J. Electr. Electron. Eng..

[87] Panno S., Davino S., Caruso A.G., Bertacca S., Crnogorac A., Mandi´c A., Noris E., Mati´c S. (2021). A review of the most common and economically important diseases that undermine the cultivation of tomato crop in the mediterranean basin. Agronomy.

[88] O’Callaghan M. (2016). Microbial Inoculation of Seed for Improved Crop Performance: Issues and Opportunities. Appl. Microbiol. Biotechnol..

[89] John C.J., Kumar S., Ge M. (2020). Probiotic Prospects of PGPR for Green and Sustainable Agriculture. Arch. Phytopathol. Plant Prot..

[90] Danish S., Zafar-ul-Hye M. (2019). Co-Application of ACC-Deaminase Producing PGPR and Timber-Waste Biochar 1034 Improves Pigments Formation, Growth and Yield of Wheat under Drought Stress. Sci. Rep..

[91] Korir H., Mungai N.W., Thuita M., Hamba Y., Masso C. (2017). Co-Inoculation Effect of Rhizobia and Plant Growth Promoting Rhizobacteria on Common Bean Growth in a Low Phosphorus Soil. Front. Plant Sci..

[92] Ahmad M., Zahir Z.A., Asghar H.N., Arshad M. (2012). The combined application of rhizobial strains and plant growth promoting rhizobacteria improves growth and productivity of mung bean (*Vigna radiata* L.) under salt-stressed conditions. Ann. Microbiol..

[93] Htwe A.Z., Moh S.M., Soe K.M., Moe K., Yamakawa T. (2019). Effects of biofertilizer produced from *Bradyrhizobium* and *Streptomyces griseoflavus* on plant growth, nodulation, nitrogen fixation, nutrient uptake, and seed yield of mung bean, cowpea, and soybean. Agronomy.

[94] Wiggins Z., Akaeze O., Nandwani D., Witcher A. (2020). Substrate properties and fertilizer rates on yield responses of lettuce in a vertical growth system. Sustainability.

[95] Vejan P., Abdullah R., Khadiran T., Ismail S., Nasrulhaq Boyce A. (2016). Role of plant growth promoting rhizobacteria in agricultural sustainability—A review. Molecules.

[96] Moncada A., Vetrano F., Miceli A. (2020). Alleviation of salt stress by plant growth-promoting bacteria in hydroponic leaf lettuce. Agronomy.

[97] Settanni L., Miceli A., Francesca N., Cruciata M., Moschetti G. (2013). Microbiological Investigation of *Raphanus sativus* L. Grown Hydroponically in Nutrient Solutions Contaminated with Spoilage and Pathogenic Bacteria. Int. J. Food Microbiol..

[98] Stouvenakers G., Dapprich P., Massart S., Jijakli M.H. (2019). Plant Pathogens and Control Strategies in Aquaponics. Aquaponics Food Production Systems.

[99] Bjarnsholt T., Van Gennip M., Jakobsen T.H., Christensen L.D., Jensen P.Ø., Givskov M. (2010). In Vitro Screens for Quorum Sensing Inhibitors and in vivo Confirmation of Their Effect. Nat. Protoc..

[100] Defoirdt T. (2018). Quorum-Sensing Systems as Targets for Antivirulence Therapy. Trends Microbiol..

[101] Ye T., Zhou T., Li Q., Xu X., Fan X., Zhang L., Chen S. (2020). *Cupriavidus* sp. HN-2, a Novel Quorum Quenching Bacterial Isolate, Is a Potent Biocontrol Agent against *Xanthomonas campestris* pv. *campestris*. Microorganisms.

[102] Sambo P., Nicoletto C., Giro A., Pii Y., Valentinuzzi F., Mimmo T., Lugli P., Orzes G., Mazzetto F., Astolfi S. (2019). Hydroponic Solutions for Soilless Production Systems: Issues and Opportunities in a Smart Agriculture Perspective. Front. Plant Sci..

[103] Gravel V., Martinez C., Antoun H., Tweddell R.J. (2006). Control of greenhouse tomato root rot [*Pythium ultimum*] in hydroponic systems, using plant-growth-promoting microorganisms. Can. J. Plant Pathol..

[104] Paradiso R., Arena C., De Micco V., Giordano M., Aronne G., De Pascale S. (2017). Changes in leaf anatomical traits enhanced photosynthetic activity of soybean grown in hydroponics with plant growth-promoting microorganisms. Front. Plant Sci..

[105] Varia J., Kamaleson C., Lerer L. (2022). Biostimulation with phycocyanin-rich Spirulina extract in hydroponic vertical farming. Sci. Hortic..

